# Tea Drinking and Cold Feet

**Published:** 1891-03

**Authors:** 


### Tea Drinking and Cold Feet.

Mr. Jonathan Hutchinson says in the *Arch. of Surg.*, that he once advised a lady to drink more tea. "I cannot touch it," was her reply. "It makes my feet icy cold, and wet with cold perspiration." On further inquiry, she assured Mr. Hutchinson that she was quite certain of her facts, and had often tested them. She thought that the perspiration was usually of the soles chiefly. Her hands were, she thought, also made cold, but not so definitely as her feet. Mr. Hutchinson says he had long been familiar with the facts that tea made the feet cold, but did not know that cold perspiration attended it.—*Canada Lancet.*

DISGUISE FOR COD-LIVER OIL.—A mixture of equal parts of cod-liver oil and lime-water is said to be nearly tasteless, but may be made more palatable by the addition of an aromatic syrup.

